# Pediatric cryptosporidiosis: An evaluation of health care and societal costs in Peru, Bangladesh and Kenya

**DOI:** 10.1371/journal.pone.0182820

**Published:** 2017-08-23

**Authors:** Ellen R. Rafferty, Janna M. Schurer, Michael B. Arndt, Robert K. M. Choy, Eugenio L. de Hostos, David Shoultz, Marwa Farag

**Affiliations:** 1 School of Public Health, University of Saskatchewan, Saskatoon, Saskatchewan, Canada; 2 Center for One Health Research, University of Washington, Seattle, Washington, United States of America; 3 PATH, San Francisco, California, United States of America; 4 Department of Global Health, University of Washington, Seattle, Washington, United States of America; Georgia Southern University Jiann-Ping Hsu College of Public Health, UNITED STATES

## Abstract

*Cryptosporidium* is a leading cause of pediatric diarrhea in resource-limited settings; yet, few studies report the health care costs or societal impacts of this protozoan parasite. Our study examined direct and indirect costs associated with symptomatic cryptosporidiosis in infants younger than 12 months in Kenya, Peru and Bangladesh. Inputs to the economic burden model, such as disease incidence, population size, health care seeking behaviour, hospital costs, travel costs, were extracted from peer-reviewed literature, government documents, and internationally validated statistical tools for each country. Indirect losses (i.e. caregiver income loss, mortality, and growth faltering) were also estimated. Our findings suggest that direct treatment costs per symptomatic cryptosporidiosis episode were highest in Kenya ($59.01), followed by Peru ($23.32), and Bangladesh ($7.62). The total annual economic impacts for the 0–11 month cohorts were highest in Peru ($41.5M; range $0.88-$599.3M), followed by Kenya ($37.4M; range $1.6-$804.5M) and Bangladesh ($9.6M, range $0.28-$91.5M). For all scenarios, indirect societal costs far outweighed direct treatment costs. These results highlight the critical need for innovative improvements to current prevention, diagnostic and treatment strategies available in resource poor settings, as well as the need for solutions that span multiple disciplines including food and water safety, sanitation and livestock production.

## Introduction

Cryptosporidiosis is a leading cause of moderate-to-severe diarrhea (MSD) in infants from resource poor regions, and particularly, where sanitation and drinking water infrastructure are lacking [1–3]. Individuals become infected when they accidentally ingest *Cryptosporidium* spp. oocysts shed in the stool of infected people or animals, and commonly experience mild to severe diarrhea, which may last up to one month [4]. Children under two years of age and individuals with pre-existing conditions that compromise immunity (e.g. HIV/AIDS) are at highest risk of severe morbidity and mortality [2,4]. Cryptosporidiosis is closely intertwined with other chronic conditions that reduce life-long earnings potential and quality of life. These include malnutrition, reduced physical fitness, growth faltering, environmental enteropathy, and possibly, cognitive impairment [5–10].

In recent years, two multiyear studies conducted in 12 countries across three continents investigated the etiologies of pediatric diarrhea (i.e. the Global Enteric Multicenter Study (GEMS) and the Interactions of Malnutrition & Enteric Infections: Consequences for Child Health and Development project (MAL-ED) [2,3]. Although GEMS and MAL-ED differed in study design, location, and methodology, both studies highlighted cryptosporidiosis prevention as a priority for children younger than 24 months. Reducing the disease burden of this pathogen is challenging for several reasons. First, the only drug currently licensed for treatment of cryptosporidiosis by the Food and Drug Administration (FDA) is restricted to children aged 12 months and older (nitazoxanide; Alinia, Romark Laboratories, Tampa, FL, USA) [11]. Its use is also limited due to variable availability in resource poor countries, cost, and reduced efficacy in malnourished or immune compromised children [12]. Second, oral rehydration salts (ORS), the WHO-recommended standard-of-care for pediatric diarrhea, remain under-utilized in many regions, despite widespread recognition regarding their efficacy in alleviating the negative impacts of diarrhea. Third, field-based diagnostics that deliver rapid, reliable and inexpensive identification of *Cryptosporidium* spp. oocysts in stool samples do not yet exist, leading to treatment courses that focus on symptoms rather than pathogen reduction [13]. Definitive diagnosis of cryptosporidiosis is further complicated by sporadic oocyst shedding. Fourth, both symptomatic (i.e. diarrheic) and asymptomatic cases, and especially those with immune-compromising conditions, can shed infective oocysts into the environment for extended periods of time [14]. This increases the likelihood of spread to other new hosts. Lastly, *Cryptosporidium* has a low infectious dose, can survive long periods of time under a variety of environmental conditions, and is resistant to household disinfectants, such as chlorine [14]. Altogether, this highlights a critical need for diagnostic, pharmaceutical, and sanitation innovations as well as streamlined opportunities for resource poor countries to access these technologies [15].

The economic costs of cryptosporidiosis, incurred both by infected individuals and by health care systems, have not been well characterized in the peer-reviewed literature. One cost-of-illness analysis conducted after a waterborne outbreak in Milwaukee, USA highlighted the relative importance of productivity losses (64.6M USD) over medical treatments (31.7M USD), and a wide range in costs between treating mild versus severe cases (116 versus 7,808 USD, respectively) [16]. Similarly, a national study measuring direct treatment costs of waterborne pathogens in the USA reported a wide cost range depending on patient insurance type (inpatient—9,361 to 20,073 USD, outpatient -267 to 757 USD) [17]. In the resource-limited context, economic evaluations commonly report all-cause diarrhea costs, or the cost-effectiveness of introducing pathogen-specific interventions, such as rotavirus vaccine [18,19]. With the recognition that cryptosporidiosis should no longer be considered a self-limiting condition comes the realization that an economic evaluation of associated morbidity and mortality is critical to setting new health care and development priorities. The goals of this study were to characterize health care costs and societal costs associated with pediatric cryptosporidiosis in three low resource countries: Peru, Bangladesh, and Kenya.

## Methods

### Country selection

Together, GEMS and MAL-ED reported incidence of pediatric cryptosporidiosis in study sites in 12 low-resource countries in Sub-Saharan Africa, South America and South Asia. We searched PubMed and Google Scholar for literature reporting *Cryptosporidium* surveillance, health care costs, and health care seeking behaviour for each of these countries, and then ranked them within continent according to the amount of available data. We selected one country per continent in order to gain regional representation from areas that differed in relevant factors including healthcare capacity, pathogen distribution, demographics, economics, and healthcare seeking behaviour. Suitability for inclusion in our study was primarily based on two criteria: 1) adequate data, and 2) low-to-moderate cryptosporidiosis incidence, as reported by GEMS or MAL-ED.

### Economic analysis

We used both a healthcare and societal approach to measure the economic burden of cryptosporidiosis for infants aged 0–11 months in Kenya, Peru and Bangladesh. To assess the burden from a societal perspective, we considered direct medical, direct non-medical and indirect costs. Direct medical cost estimates included health practitioner fees, diagnostic tests and drug costs, including ORS. Direct non-medical costs included transportation to seek care. We considered all types of healthcare costs including out-of-pockets expenditures, government payments, as well as private and public health insurance. Indirect costs included caregiver income loss, lost lifetime earnings due to morbidity (i.e. permanent growth faltering), and mortality. All costs were converted using purchasing power parity and presented in 2016 international dollars. We discounted all future costs at a rate of 3% per year as recommended by the WHO and used a life-long time horizon [20]. The base case was built on best available estimates from a range of primary and secondary sources (Table 1). Where possible we used equivalent sources to extract country-specific data for the three countries.

**Table 1:** Parameters of the pediatric cryptosporidiosis economic burden model.

**KENYA**				
**Parameter**	**Base case**	**Low estimate**	**High estimate**	**Source**
**Disease and population estimates**				
Population (0–11 months)	1,447,995	-	-	[21,22]
Incidence MSD attributable to *Cryptosporidium* (#/person-year; 0–11 months)^1^	0.04	0.02	0.072	[2]
Diarrhea severity (% of total) - MSD - LSD^1^	- 55 - 45	- 53 - 47	- 58 - 42	[23]
**Health care seeking behaviour and direct costs**				
Diarrhea cases who seek outpatient care (%)	44	34	54	[24]
Diarrhea cases who seek inpatient care (%)^1^	18	12	25	[24]
Diarrhea cases who visit a pharmacist (%)	34	31	36	[24]
Diarrhea cases visiting a pharmacist who receive (%): - Antibiotics - ORS - Anti-motility - Zinc	- 51 - 43 - 10 - 8	-	-	[25]
Outpatient cost ($/visit)^1^	25.24	12.69	85.18	[26]
Inpatient cost ($/visit)^1^	254.89	57.77	1,621.95	[26]
Inpatient length of stay (days)	3	2	5	[27]
Drug prices (median, $) - Antibiotics - ORS - Anti-motility - Zinc	- 1.39 - 1.25 - 0.57 - 3.90	-	-	[25], G. Zwisler, Pers. comm., 2016
Outpatients who pay for travel (%)	36	-	-	[26]
Inpatients who pay for travel (%)	77	-	-	[26]
Outpatient travel cost ($)	0.0	0.0	0.35	[19]
Inpatient travel cost ($)	10.65	8.48	13.05	[28]
**Indirect costs**				
Outpatient work days lost (days)	1	0	2	[25]
Inpatient work days lost (days)^1^	3	2	5	[16,19]
Caregiver wage ($/day)	5.09	-	-	[29]
Cryptosporidiosis mortality rate per 100,000 (0–364 days^1^)	21.61	0.32	62.62	[30]
Life expectancy (years)	61.6	-	-	[21]
Labour force participation (%)	67.4	-	-	[21]
Annual income (average, $/person)	3,208.07	2,735.27	9,058.21	[31],
Stunting attributable to cryptosporidiosis (%)^1^^,^^2^	8.8	3.8	17.6	C. Valencia, Pers. comm., 2016
Stunted growth persisting into adulthood (%)^1^	22.9	10	64.6	[10]
Income lost from stunted growth (%)	20	10	30	[32]
Age of entry into the workforce (years)	15	-	-	[33]
**BANGLADESH**				
**Parameter**	**Base case**	**Low estimate**	**High estimate**	**Source**
**Disease and population estimates**				
Population (0–11 months)	3,241,239	-	-	[21,30]
Incidence MSD attributable to *Cryptosporidium* (#/person-year; 0–11 months)^1^	0.007	0.002	0.012	[2]
Diarrhea severity (% of total) - MSD - LSD^1^	- 75 - 25	- 65 - 35	- 85 - 15	[34]
**Health care seeking behaviour and direct costs**				
Diarrhea cases who seek care (%)	87.9	85.00	91.00	[34]
Health sector accessed by care seekers (%)^3^: - Informal care - Outpatient care - Inpatient care	- 73.8 - 26.3 - 1.3	- 73.8 - 5.6 - 1.3	- 80.0 - 26.3 - 14.9	[34,35]
Informal care ($/visit)^1^^,^^4^	1.47	1.24	1.71	[36]
Outpatient care ($/visit)^1^^,^^4^	12.88	9.30	19.07	[37]
Inpatient care ($/visit)^1^^,^^4^	197.20	135.94	261.76	[37]
Travel to informal care ($)	0.74	0.53	0.94	[36]
Travel to outpatient care ($)	2.02	0.99	4.03	[37]
Travel to inpatient care ($)	14.15	7.08	30.81	[37]
**Indirect costs**				
Caregiver income loss per diarrhea case ($)^1^	1.85	1.21	2.50	[36]
Cryptosporidiosis mortality rate per 100,000 (0–364 days)^1^	2.36	0.035	10.13	[30]
Annual income (average, $/person)	3,686.67	-	-	[38]
Life expectancy (years)	71.6	-	-	[21]
Labour force participation (%)	70.9	-	-	[21]
**PERU**				
**Parameter**	**Base case**	**Low estimate**	**High estimate**	**Source**
**Disease and population estimates**				
Population (0–11 months)	558,749	-	-	[22]
Diarrhea frequency (# episodes/child/year)^1^	3.52	3.0	3.9	[3,39]
Diarrhea attributable to *Cryptosporidium* (%; 0–11 months)^1^	2.6	0.6	4.1	[3]
Diarrhea severity (% of total) - Severe - Non-severe (moderate & low)	- 10.6 - 89.4	- 10.6 - 89.4	- 19.2 - 80.8	[3]
**Health care seeking behaviour and direct costs**				
Diarrhea cases seeking outpatient care (%)	39.7	37.5	42.2	[18]
Severe diarrhea cases seeking inpatient care (%)	15.1	9.2	21.4	[18]
Outpatient provider costs^1^^,^^5^: ($) - Severe - Non-severe	- 41.13 - 13.8	- 23.01 - 11.00	- 73.19 - 18.21	[18]
Inpatient provider costs^1^^,^^5^ ($)	519.68	203.90	815.96	[18]
Travel cost (average, $)	0.70	0	2.05	[40]
**Indirect costs**				
Severe & Non-severe outpatient household costs^6^($)	18.15	7.98	27.11	[18]
Inpatient household costs^6^ ($)	53.83	39.90	67.76	[18]
Caregiver income loss for those who do not seek care ($)	4.89	-	-	[41]
Cryptosporidiosis mortality rate per 100,000^1^	0.107	0.0096	0.796	[30]
Life expectancy (years)	74.5	-	-	[21]
Labour force participation (%)	76.3	-	-	[21]
Annual income (average, $/person)	13,838.99	-	-	[38]

All costs are presented in 2016 international dollars; base case determined from best estimates available; low and high estimates provided for pre-selected variables included in sensitivity analysis; MSD = Moderate to Severe Diarrhea, LSD = Less Severe Diarrhea, ORS = Oral Rehydration Salts

^1^Variables included in one-way sensitivity analysis

^2^Stunting and age of entry into the workforce parameters were uniform across countries

^3^Does not add up to 100% because individuals can seek more than one form of care

^4^These costs include diagnostics, pharmaceuticals and provider care

^5^Provider costs include average outpatient and inpatient visit costs and drug costs

^6^Household costs include income loss costs and out-of-pocket expenditures

We conducted one-way sensitivity analyses on up to 10 key variables per country. Variables were selected either because they had the potential to substantially impact overall costs or because there was a high degree of uncertainty in the parameter estimate. We varied the discount rate between zero and five percent. As well, we included a scenario analysis where we varied all growth faltering parameters simultaneously. Furthermore, we conducted best and worst case scenario analysis to determine the projected highest and lowest economic impact of pediatric cryptosporidiosis for each country under our current assumptions.

### Disease and population estimates

We obtained age-specific population size estimates from the most recent in-country census for Peru (2007), Bangladesh (2011), and Kenya (2009) and inflated them to 2016 values using World Bank population growth estimates [21]. The number of age-specific MSD cases attributable to *Cryptosporidium* in Kenya and Bangladesh were derived from GEMS [2]. As GEMS only presented the incidence for MSD attributable to *Cryptosporidium*, we combined these parameters with the proportion of MSD to LSD cases to calculate the total number of diarrhea cases attributable to *Cryptosporidium* for the 0–11 month age group (Eq 1).

$$\begin{array}{l} \text{Total }\#\text{ cryptosporidiosis cases}\\ =\;\left(\text{Population }\times \text{ Incidence MSD caused by }Cryptosporidium\right)\\ +\;\left\{\left[\frac{(\text{Population }\times \text{ Incidence MSD caused by }Cryptosporidium\text{)}}{\text{Proportion MSD cases of total diarrhea cases}}\right]\times \text{ Proportion LSD cases of total diarrhea cases}\right\} \end{array}$$ (1)

For Peru, we calculated the number of age-specific cases using the percentage of diarrheal cases attributable to *Cryptosporidium* and the frequency of diarrhea episodes per child per year reported by MAL-ED [3], as well as population estimates (Eq 2).

$$\begin{array}{l} \text{Total }\#\text{ cryptosporidiosis cases}\\ =\left(\text{Population }\times \text{ Frequency of diarrhea episode per child year}\right)\\ \times \;\%\text{ diarrhea attributable to }Cryptosporidim\text{ for population} \end{array}$$ (2)

### Direct medical and non-medical costs

Medical costs were calculated using country-specific estimates of caregiver health care seeking behaviour for cases of diarrhea. To estimate health care seeking behaviour for Kenya and Bangladesh we used Health Utilization and Attitudes Surveys (HUAS) that were completed prior to the GEMS study. The HUAS qualify and quantify the health sectors accessed by caregivers of childhood diarrhea cases that seek health care (e.g. informal care, pharmacist, outpatient and inpatient care) [24,34]. For Peru, we extracted outpatient and inpatient utilization rates from a recently published cost-effectiveness analysis evaluating rotavirus vaccine [18]. Costs of medical care and transportation were derived from a number of country-specific sources, including general reports on in-country health care costs [26], previous economic analyses [18,37], pharmacy data and household surveys [25,36,41] (Table 1). Our calculation of direct medical and non-medical costs for Bangladesh is presented in Eqs 3 and 4, respectively. Additional information on these calculations, as well as specific formulas for Peruvian and Kenyan costs are available in S1, S2, S3 and S4 Tables.

$$\begin{array}{l} \text{Direct medical costs }\left(\text{Bangladesh}\right)\\ =\;\left(\#\text{ who seek informal care }\times \text{ Avg cost per informal care visit}\right)\\ +\;\left(\#\text{ who seek outpatient care }\times \text{ Avg cost per outpatient care visit}\right)\\ +\;\left(\#\text{ who seek inpatient care }\times \text{ Avg cost per inpatient care visit}\right) \end{array}$$ (3)

$$\begin{array}{l} \text{Direct non}-\text{medical costs }\left(\text{Bangladesh}\right)\\ =\;\left(\#\text{ who seek informal care }\times \text{ Avg travel cost for informal care}\right)\\ +\;\left(\#\text{ who seek outpatient care }\times \text{ Avg travel cost for outpatient care}\right)\\ +\;\left(\#\text{ who seek inpatient care }\times \text{ Avg travel cost for inpatient care}\right) \end{array}$$ (4)

### Indirect costs

We included three different types of indirect costs: (1) caregiver income loss costs; (2) mortality costs; and (3) growth faltering costs. Caregiver costs were extracted from in-country household interviews [19,28,36,41], which estimated the amount of income (Bangladesh and Peru) or work time (translated into income- Kenya) lost to care for a child with diarrhea. Country-specific mortality costs were calculated using Eq 5, derived from [33]. To estimate the average number of work years, we assumed that an individual would enter the workforce and start earning wages at 15 years of age; therefore, we had a delayed earnings stream starting at 15 years and ending at the country-specific life expectancy. To calculate the net present value of work years, earnings were discounted at a rate of 3% per year.

$$\begin{array}{l} \text{Income loss from cryptosporidiosis mortality}\\ =\;\sum_{\text{n}=15\text{ to LE}}\frac{\left(\text{Mortality rate }\times \text{ Country}-\text{specific annual earnings }\times \text{ Population }\times \text{ Labour force participation}\right)}{{\left(1\;+\text{ Discount rate}\right)}^{\text{n}}} \end{array}$$ (5)

Where:

LE = life expectancy in a given country

n = value between 15 (year earning begins) and life expectancy (year earnings end)

Finally, we assumed that a fraction of infants with acute growth faltering attributable to *Cryptosporidium* infection would not experience catch up growth, and that permanent stunting would impact future potential earning. Our estimates of these factors were based on a longitudinal birth cohort of children in Bangladesh monitored for growth faltering and cryptosporidiosis [10]. To determine the percentage of children with stunted growth due to cryptosporidiosis, we calculated the percentage difference of children stunted with and without *Cryptosporidium* infection (i.e. 8.8%). We assumed that only children categorized as severely stunted (Height-for-age- adjusted z-score (HAZ) ≤-3) would remain stunted into adulthood (i.e. 23.8% of the cohort). Country-specific income loss due to growth faltering was calculated as follows:

$$\begin{array}{l} \text{Income loss to stunting}\\ =\;\sum_{\text{n}=15\text{ to LE}}\frac{\begin{array}{l} \text{Number infected with }Cryptosporidium\;\times \;\%\text{ stunting attributable to }Cryptosporidium\;\times \\ \%\text{ of permanent stunting }\times \;(\text{Country}-\text{specific average annual income }\times \;\%\text{ income loss due to stunting})\\ \times \text{ Labour force participation} \end{array}}{{\left(1\;+\text{ Discount rate}\right)}^{\text{n}}} \end{array}$$ (6)

Where:

LE = life expectancy in a given country

n = value between 15 (year earning begins) and life expectancy (year earnings end)

## Results

### Base case analysis

Overall, Peru had the highest incidence of cryptosporidiosis in children in the first year of life (0.091 cases/person-year), followed by Kenya (0.084 cases/person-year) and Bangladesh (0.0093 cases/person-year); however, Bangladesh had the largest cohort size for that age group (3,241,239 children). Kenya had the highest number of cryptosporidiosis cases, despite having less than half the number of infants in the 0–11 month cohort compared to Bangladesh. In the first year of life, mortality attributable to cryptosporidiosis was highest in Kenya (21.61 per 100,000 children), followed by Bangladesh (2.36 per 100,000) and lowest in Peru (0.11 per 100,000). Health care seeking behaviour also differed by country with variations in the type of care sought (formal versus informal), and the percentage of caregivers who accessed each type of care for their children.

Our evaluation of health care costs incurred by cryptosporidiosis patients suggests that the direct cost per case varied from $23.32 (range $9.97-$60.84) and $7.62 (range $3.45-$4.12) in Peru and Bangladesh to $59.01 (range $12.5-$454.80) in Kenya (Table 2). This difference was primarily caused by the higher costs of inpatient care in Kenya and the larger percentage of infected individuals seeking inpatient care compared to informal or outpatient care. The indirect costs per case in Peru ($812.90) were more than double those in Kenya or Bangladesh. This is explained by the higher average annual income and life expectancy in Peru, both of which prolong the burden of disability or mortality.

**Table 2:** Total costs associated with pediatric cryptosporidiosis (2016 international dollars).

	Kenya	Bangladesh	Peru
**Cryptosporidiosis cases (#/year)** - Number deaths - Number stunted	121,105 - 312 - 2,443	30,251 - 76 - 610	51,085 - 1 - 1,030
**Total direct costs ($)** - Outpatient - Inpatient - Travel - Other^1^	7,146,611 - 1,344,729 - 5,556,374 - 178,833 - 66,67	230,564 - 90,045 - 77,554 - 34,098 - 28,867	1,191,343 - 707,603 - 468,949 - 14,791 - 0
**Total indirect costs ($)** - Caregiver income loss - Stunted growth - Mortality	30,246,389 - 1,292,238 - 17,652,388 - 11,301,563	9,385,932 - 49,279 - 5,728,064 - 3,608,590	40,331,497 - 146,769 - 40,068,197 - 116,531
**Costs per case ($)** - Direct - Indirect	- 59.01 - 308.77	- 7.62 - 317.88	- 23.32 - 812.80
**Best case scenario ($)**	1,606,495	277,312	883,807
**Worst case scenario ($)**	804.5M	91.5M	599.3M
**Total societal costs ($)**	**37.4M**	**9.6M**	**41.5M**

^1^Other includes costs for drugs, informal care and insurance

When all types of costs were considered, Peru had the highest total annual economic burden due to cryptosporidiosis in infants aged 0–11 months ($28.5M), even though it had the smallest cohort population and the lowest mortality rate. Annual costs for symptomatic *Cryptosporidium* infections were lower in Kenya ($24.2M) and Bangladesh ($5.5M). Indirect costs contributed the largest portion of economic burden caused by pediatric cryptosporidiosis, accounting for 80.9%%, 97.1% and 97.6% of costs in Kenya, Peru and Bangladesh respectively. Growth faltering was responsible for the greatest impact on overall costs in all three countries. Of the direct costs, inpatient costs in Kenya had the greatest economic impact; however, in Peru and Bangladesh outpatient care was estimated to be the principal direct cost.

### Sensitivity analysis

**Fig 1:**
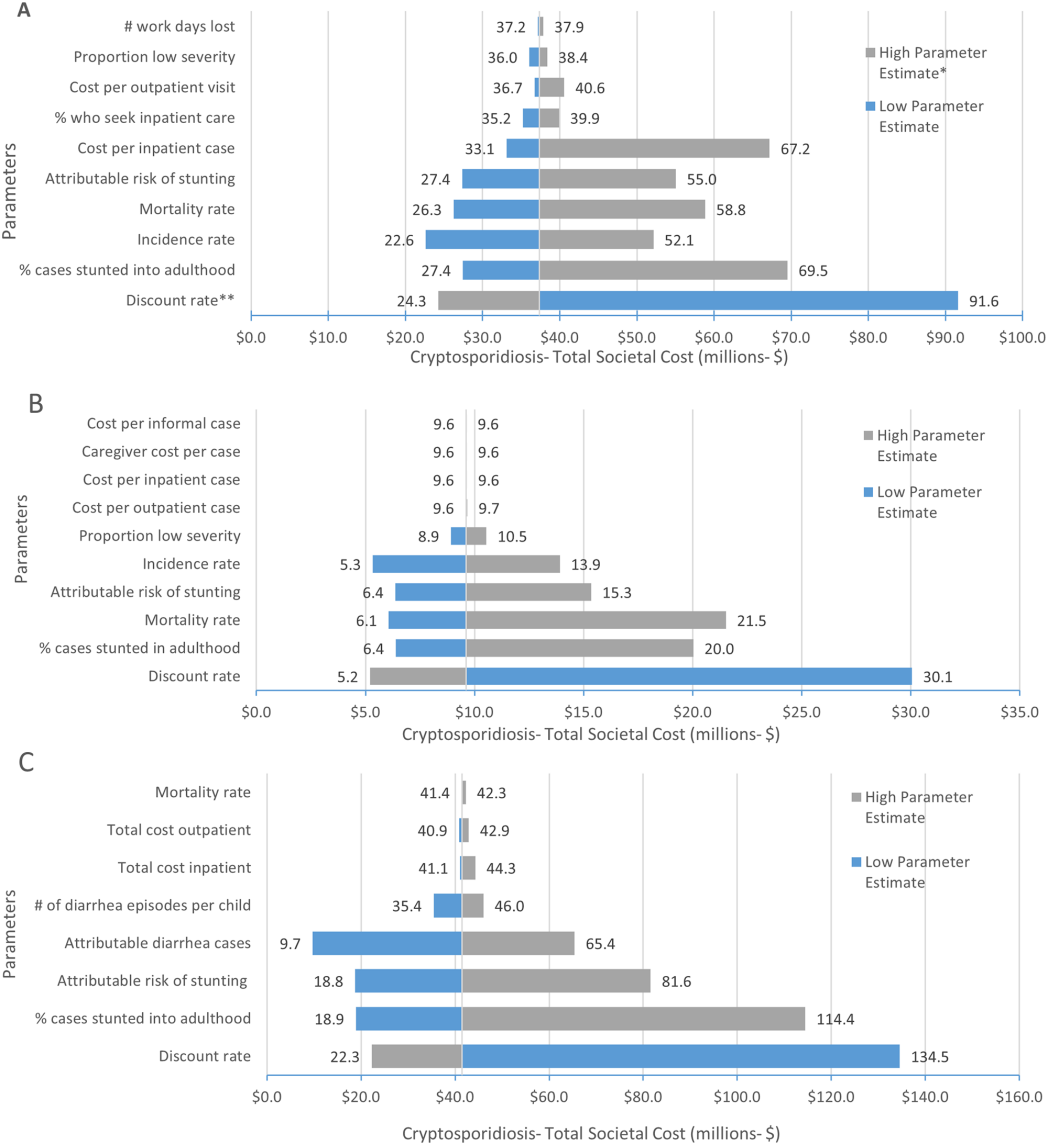
Sensitivity analysis tornado diagrams for the evaluation of healthcare and societal costs associated with cryptosporidiosis in infants aged 0–11 months in three countries. (A) Kenya. (B) Bangladesh. (C) Peru.

Our analysis showed a wide variation in best and worst case scenario estimates for the economic burden of cryptosporidiosis across the three countries. The difference in best and worst case ranges were largely driven by uncertainty in disease incidence, as well as a scarcity of data quantifying growth faltering attributable to *Cryptosporidium* infection and the frequency of faltered growth persisting into adulthood (Fig 1). The significant impact and uncertainty of growth faltering was further confirmed in the scenario analysis, where varying all the growth faltering parameters at once showed large variations in the overall economic burden of cryptosporidiosis (Peru: $5.2M- 342.2M, Bangladesh: $4.4M-52.6M, Kenya $21.4M-168.9M). Discount rate was the most influential parameter in all three countries. Overall estimates were also sensitive to country-specific mortality and incidence rates. Uncertainty relating to direct costs had little influence on overall societal costs, except in Kenya, where a wide range in reported inpatient costs is known to occur.

## Discussion

In 2016 the United Nations member states set out to dramatically improve global health and development outcomes by defining 17 priority areas. Sustainable Development Goal 3 aimed to reduce the mortality rate of children younger than 59 months to at least 25 deaths per 1000 live births before 2030. Diarrhea is a key contributor to morbidity and mortality in resource-limited settings, and despite significant achievements in reducing incidence over the last decade, it remains a priority area for decreasing pediatric mortality [42]. In recent years, the GEMS and MAL-ED findings have shifted how the global community prioritizes pathogens related to diarrhea by highlighting the relative importance of C*ryptosporidium* spp. in causing diarrhea in infants under 24 months [2,3]. Our economic analysis of cryptosporidiosis in Peru, Bangladesh and Kenya demonstrated that C*ryptosporidium* causes a large burden both at the household level and for society as a whole, which has negative consequences for achieving other development goals, such as poverty alleviation. The large societal costs combined with the negative consequences of infection underscore the importance of *Cryptosporidium*-specific technology innovations and policy interventions suitable for resource-limited areas.

Direct and indirect medical costs varied among the three study locations. Our sensitivity analysis suggested that direct medical costs made only a small contribution to the total societal costs relative to indirect costs in the three target countries. The relatively large impact of indirect costs on the overall economic burden of cryptosporidiosis helps explain why discounting factor was the most influential parameter in all three countries. Our results in Kenya were supported by an economic evaluation of GEMS participants, which found that direct medical costs represented only 11% of the total economic burden associated with pediatric diarrhea in, Kenya [43]. However, direct costs represented the majority (52%) of the total burden for GEMS participants in Bangladesh [36]. Our findings for Bangladesh likely conflicted with those from GEMS due to our inclusion of growth faltering and mortality, which substantially increased the estimation of indirect costs. It remains unclear how our Peru results compare to other studies, as the MAL-ED authors have not published an economic assessment, and we were unable to find other comparable evaluations in the peer-reviewed literature. Our estimates of per episode direct medical costs were generally higher than those reported by GEMS.

We estimated that direct costs per *Cryptosporidium* infection were highest in Kenya ($59.01), followed by Peru ($23.32) and Bangladesh ($7.62). The higher direct costs observed in Kenya are mainly attributable to higher outpatient and inpatient utilization rates in Kenya, relative to Bangladesh or Peru. GEMS reported mean costs per diarrheal episode as 1.82 USD in Bangladesh [35] and 6.24 USD in Kenya [43]. This difference in estimates is partially due to differences in currency (international $ vs USD); however, it might also indicate that treating cryptosporidiosis is more costly than treating other pathogens responsible for childhood diarrhea. Moreover, our analysis aimed to capture all costs associated with treating cryptosporidiosis, including government and private care costs, while GEMS reported specifically on household costs. Regardless, these estimates signify a considerable burden for those living below the international poverty line (1.25 USD per day), especially given the recurrent nature of this disease for children in resource-poor regions [15]. Furthermore, comparing the direct and indirect costs per case to the GDP per capita of Peru ($6,027), Kenya ($1,377) and Bangladesh ($1,212) suggests that cryptosporidiosis has a major financial impact on individuals and families dealing with this disease [21]. The overall direct care costs represented only a small fraction of each country’s health care spending (Peru- 0.011%, Bangladesh- 0.005%, Kenya-0.200%), suggesting that more health funding may be need to control this pathogen, particularly as indirect costs place a large burden on families and governments [21].

Our economic evaluation focused on human cases of cryptosporidiosis; however, *Cryptosporidium* is a zoonotic protozoan, with the potential to inflict losses on other industries, such as cattle production. Approximately 26 *Cryptosporidium* species are currently recognized, most of which exhibit high host specificity and generally cross the species barrier only when they encounter immune compromised individuals. Humans are the primary definitive host for *C*. *hominis*, but are also infected by *C*. *parvum*, which is a zoonotic species infective to numerous nonhuman hosts. Molecular analysis of a subset of GEMS participants with MSD identified *C*. *hominis* as the most common *Cryptosporidium* species, indicating that human-to-human transmission was a greater cause of pediatric infection than zoonotic transmission [44]. Other studies of children under five confirmed this finding in Kenya [45], Peru [46], and Bangladesh [10], also reporting prolonged oocyst shedding and a greater severity and diversity of symptoms (e.g. vomiting, fever, nausea, abdominal pain, malaise, and diarrhea) in those infected with *C*. *hominis* versus other species [45,46]. Together, these findings emphasize the need for improved sanitation in areas experiencing high burdens of disease, particularly in locations with overcrowding or high population densities and where children regularly interact (e.g. childcare facilities).

Our analysis aimed to quantify and describe country-specific practices related to seeking health care, diagnosing, and treating children with cryptosporidiosis symptoms. It is important to note that many of the reported norms did not align with practices endorsed by the medical community, and that this paper does not report health care costs in settings where all children receive evidence-based care. In Peru, for example, antibiotics are commonly prescribed to treat diarrhea cases in lieu of diagnostic tests paired with pathogen specific treatment. Only 31% of Peruvian children under five with diarrhea receive ORS, and nitazoxanide use is uncommon [47]. In Bangladesh, most pediatric cases receive ORS (84%), often in combination with zinc and/or antibiotics [48]; whereas, many Kenyan parents prefer herbal remedies, or anti-motility syrups and antibiotics to accelerate symptom cessation [49]. Care seekers in the three countries accessed a variety of providers, including traditional healers, faith healers, community health workers, pharmacists, physicians and nurses. Furthermore, household costs differed according to the health sector utilized (public, private, or non-government organization) and country-specific coverage provided by governments and insurance schemes to alleviate health care costs. For this reason, we report overall health costs, which are paid out by individuals and/or governments, accordingly.

A critical gap currently exists in our understanding of the societal, pathological, and economic impacts of cryptosporidiosis in high-risk groups and geographic locales. We limited our evaluation to one high-risk age cohort in each of the three target countries because important limitations currently exist in data related to disease incidence, social determinants of health, health care seeking behaviour, cognitive delay and long-term sequelae (e.g. growth faltering). Pediatric cryptosporidiosis is a complex illness with risk factors that relate to the immune status of hosts, to species differences in the parasite, and to social determinants of health that impact exposure and recovery (e.g. education, sanitation, poverty). In particular, it remains unclear whether short-term stunting observed in children younger than five persists into adulthood, and whether other sequelae associated with diarrhea (e.g. cognitive delay, malnutrition, impaired physical fitness) are also associated with cryptosporidiosis [5,6,8,10]. Improvements in detecting pathogens shed by diarrheic children have facilitated the investigation of acute health manifestations; however, long-term pathogen-specific sequelae, such as growth faltering, malnutrition, impaired physical fitness, and cognitive delay remain under-investigated.

Other differences relating to demographics were not captured by our analysis, including disparities between rural and urban care seekers, and willingness to seek care for male versus female children. In the absence of more comprehensive data, our model used cryptosporidiosis incidence estimates established for rural districts in Kenya, Peru, and Bangladesh in combination with care seeking and cost information established for both urban and rural areas. Improvements to available data are needed in order to better characterize human, veterinary, and societal costs of cryptosporidiosis. Furthermore, the complexity of disease transmission along with poor treatment options and the complex interaction of this disease with other health compromising conditions (e.g. malnutrition and HIV/AIDS) make mitigation of *Cryptosporidium* infection a challenging problem. Successful mitigation will likely require a multi-sectoral One Health type approach, integrating input from experts in water, sanitation, and hygiene (WASH), food safety, livestock production, education, and family health (especially maternal and child health, and nutrition) [33]. In particular, such efforts should be evaluated for cost-effectiveness in resource-limited contexts.

## Supporting information

S1 Table Cost calculations for cryptosporidiosis in children 0–11 months-old in Kenya. (XLSX)

S2 Table Cost calculations for cryptosporidiosis in children 0–11 months-old in Bangladesh. (XLSX)

S3 Table Cost calculations for cryptosporidiosis in children 0–11 months-old in Peru. (XLSX)

S4 Table Direct medical and non-medical costs equations to assess the cryptosporidiosis burden in 0–11 month-olds (Bangladesh, Kenya and Peru). (XLSX)
